# Bronchiolitis obliterans syndrome associated with an immune checkpoint inhibitor in a patient with non-small cell lung cancer

**DOI:** 10.1016/j.rmcr.2023.101824

**Published:** 2023-02-26

**Authors:** Kenichiro Takeda, Hideki Miwa, Masato Kono, Ryutaro Hirama, Yuiko Oshima, Yasutaka Mochizuka, Akari Tsutsumi, Yoshihiro Miki, Dai Hashimoto, Hidenori Nakamura

**Affiliations:** a Department of Pulmonary Medicine, Seirei Hamamatsu General Hospital, Hamamatsu, Japan; b Department of Respirology, Graduate School of Medicine, Chiba University, Chiba, Japan; c Second Division, Department of Internal Medicine, Hamamatsu University School of Medicine, Hamamatsu, Japan

**Keywords:** Non-small cell lung cancer, Bronchiolitis obliterans syndrome, Immune checkpoint inhibitor, Pembrolizumab, Immune-related adverse events

## Abstract

A 75-year-old woman was admitted to our hospital with progressive dyspnea 7 months after second-line treatment with pembrolizumab for advanced non-small cell lung cancer. Chest radiography revealed hyperinflation in both lung fields, and pulmonary function tests revealed severe obstructive dysfunction without bronchodilator reversibility. There were no identifiable causes such as infections or autoimmune diseases. Therefore, bronchiolitis obliterans syndrome associated with immune checkpoint inhibitors was clinically diagnosed. Pembrolizumab was discontinued, but the respiratory dysfunction was irreversible and resulted in death. Bronchiolitis obliterans syndrome is an extremely rare but potentially severe adverse event associated with immune checkpoint inhibitor-related lung disease.

## 1 Introduction

Immune checkpoint inhibitors (ICIs) activate and sustain antitumor immunity by blocking co-inhibitory molecules that promote tumor immune escape [1]. Consequently, ICI administration as a cancer treatment is rapidly increasing, both as monotherapy and in combination with other cancer treatments [2]. However, ICIs can induce autoimmune inflammatory disease-like side effects called immune-related adverse events (irAEs) [3]. irAEs can affect almost all organs, such as the lungs; this occurs in 5.0%–6.0% of cases [1,4,5] and presents as organizing pneumonia, hypersensitivity pneumonitis, and nonspecific interstitial pneumonitis.

Bronchiolitis obliterans (BO) is an irreversible obstructive airway disease with various potential causes. Many studies have described lung or bone marrow transplantation-associated BO. On the other hand, non-transplantation-associated BO cases (associated with inhalational toxins, drugs, autoimmune disease, and infections) have been reported [6]. However, BO associated with ICIs is extremely rare.

Although histologic confirmation is required for the diagnosis of BO, transbronchial biopsy specimens often are not sufficiently sensitive [7]. Therefore, a clinical description of BO, termed BO syndrome (BOS), has been proposed and defined according to pulmonary function changes rather than histology [8].

We describe a case of BOS that occurred during pembrolizumab treatment for postoperative recurrent metastatic lung adenocarcinoma.

## 2 Case presentation

A 75-year-old woman who received pembrolizumab (200 mg/body every 3 weeks) for 7 months (11 cycles) as treatment for postoperative recurrent lung adenocarcinoma was admitted to our hospital because of progressive dyspnea. The patient never smoked and had a medical history of right lower lobectomy with lymph node resection for lung adenocarcinoma (pT1aN0M0, pStage IA) 16 years before presentation. Twelve months before admission, the patient was diagnosed with recurrent lung adenocarcinoma harboring bone metastasis, which was epidermal growth factor receptor exon 19 deletion-positive with a 50% programmed death-ligand 1 (PD-L1) expression level. First-line treatment with osimertinib (80 mg/day) was discontinued because of drug-induced interstitial lung disease (ILD); most ground-glass opacities improved, but only after discontinuing the medication. At that time, she did not exhibit respiratory failure.

**Fig. 1:**
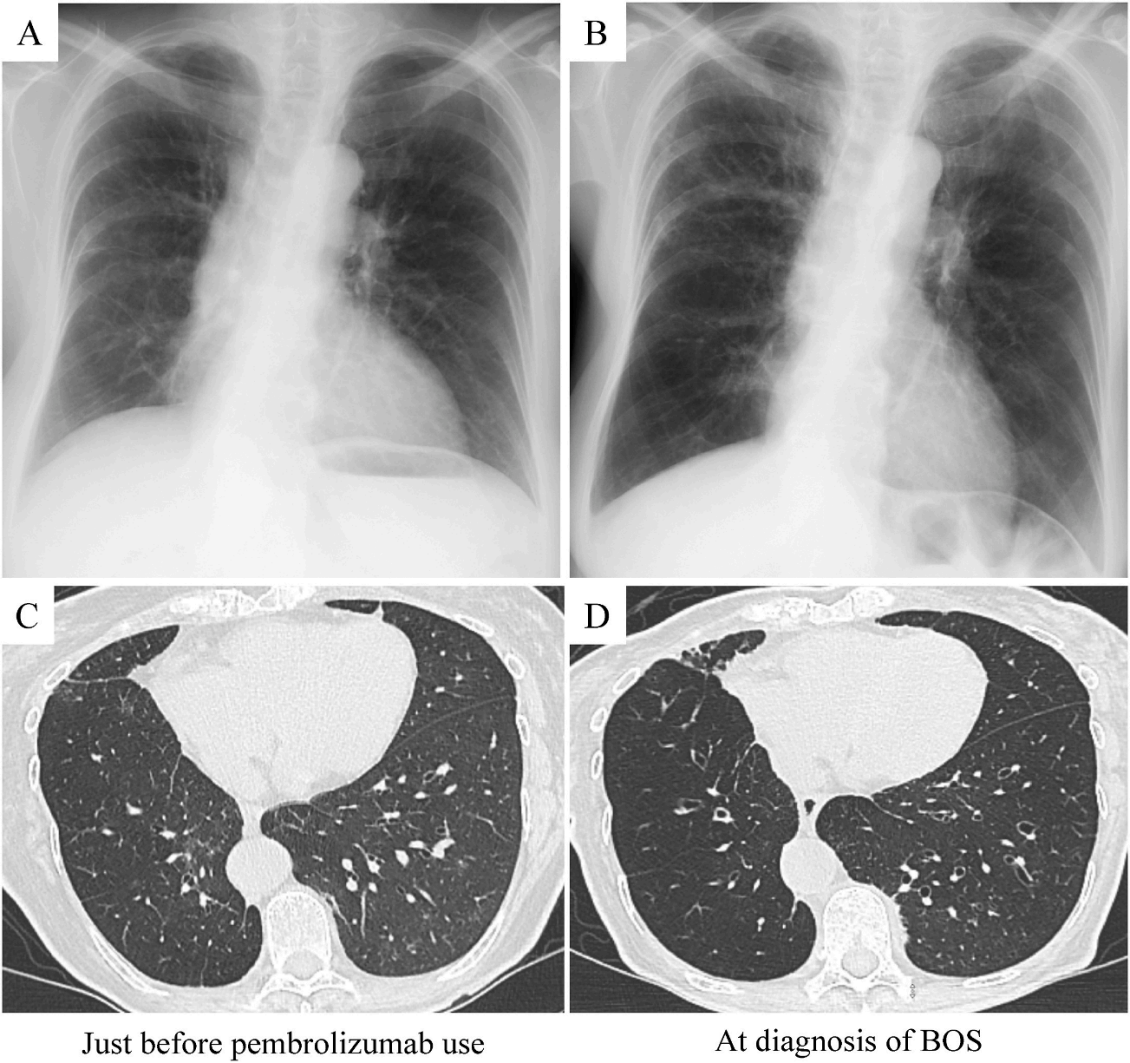
Compared to the chest radiograph results obtained at the beginning of pembrolizumab treatment (A), the radiograph results obtained at admission (B) show hyperinflation in the right lower field. Compared with the image obtained just before pembrolizumab use (C), the chest inspiratory computed tomography image at the time of the BOS diagnosis shows reduced vascular caliber and lung density in the right lower lobe and dilated airways in the both lower lobes (D).

**Fig. 2:**
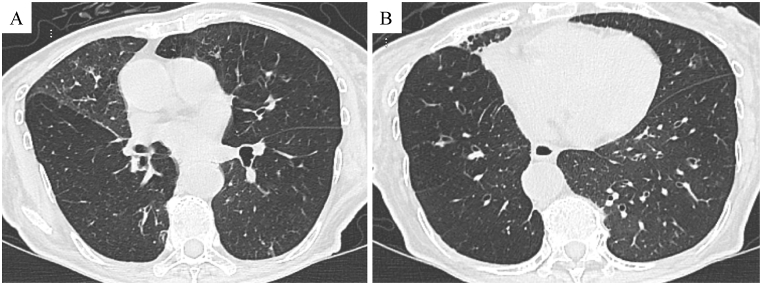
Chest expiratory computed tomography (CT) image shows decreased lung field density in the right lower lobe compared with the right middle lobe (A). Similar to the inspiratory CT results, bronchial dilatation was observed in the lower lobes of both lungs (B).

**Fig. 3:**
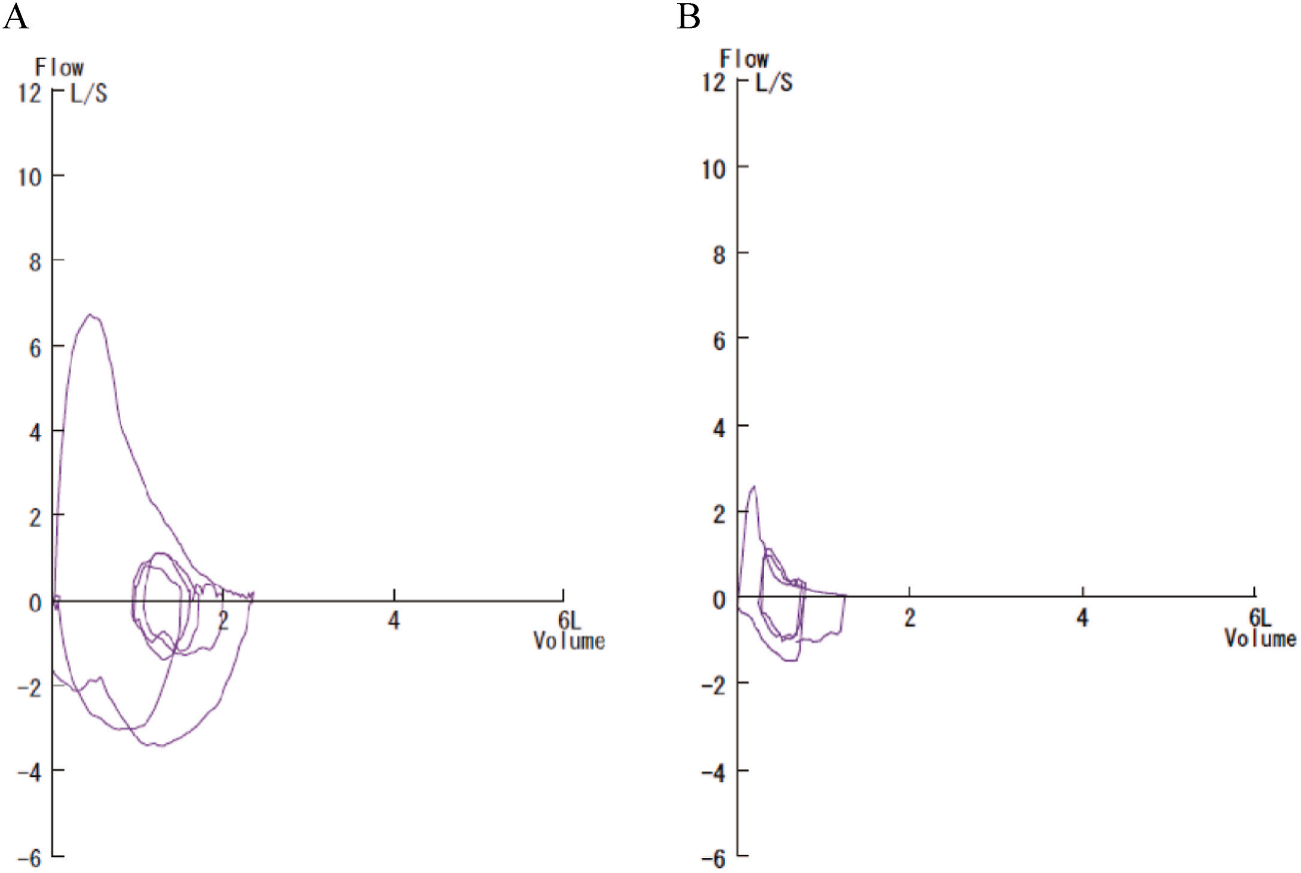
The flow-volume curve 13 years before bronchiolitis obliterans syndrome (BOS) was diagnosed (3 years after right lower lobectomy) (A) and at the time of the BOS diagnosis (B). Expiratory flow limitations were observed at the time of the BOS diagnosis.

At the time of admission, the results of physical examinations, including chest auscultation, were unremarkable. The patient's body temperature was 36.6 °C. Her blood pressure was 110/60 mm Hg, heart rate was 83 bpm, and oxygen saturation level was 92%. Laboratory tests showed a normal white blood cell count and normal C-reactive protein and D-dimer levels. A blood gas analysis showed a partial pressure of arterial oxygen (PaO_2_) of 58.9 Torr and partial pressure of arterial carbon dioxide (PaCO_2_) of 44.9 Torr without supplemental oxygen (i.e., room air). Compared to the findings at the beginning of pembrolizumab treatment (Fig. 1A), chest radiography showed hyperinflation in the right lower field (Fig. 1B). Compared to the images obtained just before pembrolizumab use (Fig. 1C), chest inspiratory high-resolution computed tomography (CT) showed reduced vascular caliber and lung density in the right lower lobe and bronchial dilatation in the bilateral lower lobes (Fig. 1D). Expiratory CT showed decreased lung field density in the right lower lobe compared with that in the right middle lobe (Fig. 2A) and dilated peripheral bronchi in the bilateral lower lobes (Fig. 2B). There was no evidence of pulmonary embolism. Pulmonary function tests revealed a new restrictive and obstructive disorder (Table 1), and the flow-volume curve showed expiratory flow limitations (Fig. 3B). Bronchodilator reversibility was not observed (Table 1). After excluding bronchial asthma, chronic obstructive pulmonary disease, and diffuse panbronchiolitis, the patient was diagnosed with BOS. No other causes of BOS were identified (e.g., autoimmune disease, infection, organ transplantation, other medications, or inhalational toxins). Therefore, the patient was diagnosed with pembrolizumab-associated BOS.

**Table 1:** Pulmonary function test results before, during, and after the diagnosis of BOS.

	13 years before^a^	At the time of diagnosis		16 months after
		Pre-BD	Post-BD	
VC, L (%)	2.34 (95.9)	1.45 (65.9)	–	1.38 (63.0)
FVC, L (%)	2.36 (96.7)	1.37 (62.3)	1.43 (65.0)	1.33 (60.7)
FEV_1_, L (%)	1.88 (94.9)	0.64 (40.8)	0.68 (43.4)	0.54 (34.8)
FEV_1_/FVC, %	79.7	46.7	47.5	40.6
TLC, L (%)	–	3.60 (98.6)	–	3.63 (97.8)
RV, L (%)	–	2.11 (131.9)	–	2.36 (138.8)

BD, bronchodilator; BOS, bronchiolitis obliterans syndrome; FVC, forced vital capacity; FEV_1_, forced expiratory volume in 1 second; RV, residual volume; TLC, total lung capacity; VC, vital capacity.

a The test was performed 3 years after the right lower lobectomy.

Pembrolizumab was discontinued, and the patient was treated with inhaled corticosteroids (fluticasone propionate 500 μg/day), long-acting β_2_ agonists, low-dose erythromycin (400 mg/day), a leukotriene antagonist, and home oxygen therapy. However, dyspnea progressed, and the patient's respiratory function did not improve 16 months after the onset of BOS (Table 1). The blood gas analysis showed a PaO_2_ of 80.4 Torr and a PaCO_2_ of 54.1 Torr with 2 L/min of oxygen. Emaciation progressed with the appearance of small metastases in both lung fields. The patient died of progressive respiratory failure at 21 months after the BOS diagnosis.

## 3 Discussion

This report describes a patient who developed BOS after pembrolizumab treatment for postoperative recurrence of lung adenocarcinoma. The patient presented with dyspnea and type I respiratory failure 7 months after starting pembrolizumab treatment. Respiratory function tests showed a new obstructive disorder, and chest radiography revealed hyperinflation. The possibility of other obstructive pulmonary diseases was ruled out; no other factors could have caused BOS. Therefore, we diagnosed pembrolizumab-induced BOS.

Programmed death-1 (PD-1) is expressed on activated B and T cells and binds to its ligand (PD-L1) on tumor cells, allowing them to evade the immune response [5]. Pembrolizumab is a selective monoclonal antibody against PD-1 that blocks the transmission of inhibitory signals to maintain T-cell activation and restore antitumor effects. irAEs are widely recognized and related to exaggerated immune system activation [9], affecting nearly all organs, including the lungs [1,4,5]. Delaunay et al. reported that organizing pneumonia, hypersensitivity pneumonitis, and nonspecific interstitial pneumonitis are common irAEs, but they did not mention BO [10]. During a phase II trial of nivolumab plus ipilimumab for patients with esophageal squamous cell carcinoma, one treatment-related death attributable to BO was reported [11]. However, it is unclear whether these drugs were directly involved in the onset of BO. Moreover, only one report by Blanchard et al. has described BO associated with ICIs. They reported a case of BO in a 69-year-old woman with lung squamous cell carcinoma who presented with dyspnea at the time of the seventh pembrolizumab cycle. BO was diagnosed based on a new severe obstructive disorder, mosaic attenuation on chest CT, and a lack of response to corticosteroids. Similar to our case, a lung biopsy was not performed because of a severe respiratory disorder [9]. We believe that our case is extremely rare and provides important information about respiratory disorders after ICI treatment.

BO is a rare but serious disease characterized by progressive and irreversible airway obstruction induced by injury to the respiratory and terminal bronchioles attributable to various causes [6]. BO presents as a pulmonary manifestation of chronic graft-versus-host disease after hematopoietic cell transplantation (HSCT) and as a noninfectious form of chronic lung allograft dysfunction after lung transplantation [6]. Additionally, exposure to inhaled toxins (e.g., sulfur mustard and nitrogen oxide), drugs (e.g., penicillin), autoimmune disorders (e.g., rheumatoid arthritis), and infections (e.g., adenovirus, measles virus, or mycoplasma) can induce BO [6,12]. Although a BO diagnosis is based on histological changes, transbronchial biopsies are insufficiently sensitive [7]. Therefore, experts endorse a clinical description of BO, termed BOS, that is defined by pulmonary function changes rather than histological changes [8]. The International Society for Heart and Lung Transplantation proposed a clinical description of BOS after lung transplantation based on changes in mid-expiratory flow rates (forced expiratory flow 25%–75%) or forced expiratory volume in 1 second (FEV_1_) from baseline [8]. The National Institutes of Health defined the clinical diagnosis of BOS after HSCT as pulmonary function abnormalities (FEV_1_/vital capacity <70% and FEV_1_ <75% with a predicted 10% decline over the course of <2 years) [13]. Despite the differences in the BOS criteria among the causative diseases, rapid FEV_1_ decline is important for the diagnosis of BO. In our case, 13 years elapsed between the lung function tests before and after BOS. However, type I respiratory failure and hyperinflation suddenly appeared; these were not observed before pembrolizumab use. Therefore, the severe obstructive disorder likely appeared after drug use, contributing to shortness of breath. Pulmonary function tests did not show bronchodilator reversibility, and the chest CT did not show diffuse micronodules, bullae, or emphysema. Additionally, the patient did not respond to inhaled corticosteroids, long-acting β_2_ agonists, or leukotriene antagonists. Therefore, we diagnosed BOS based on these results and the clinical course.

T cells are assumed to have an important role in the pathogenesis of ICI-induced BO. ICI can cause immune effector and T-cell deregulation and peri-bronchial inflammation [2,10]. In mouse BO models, CD4^+^ and CD8^+^ T cells are required for BO development [14,15]. These cells contribute to inflammation around the airway, inducing epithelial cell injury and the loss of epithelial progenitor cells [14,15]. Epithelial injury leads to fibrotic phenomena and bronchial obstruction through the epithelial-mesenchymal transition [16]. However, the mechanisms of ICI-induced BO have not been directly elucidated, and further investigation is required.

Administering ICIs to patients with pre-existing ILD is associated with a high risk of ICI-induced pneumonitis. A meta-analysis revealed that ground-glass attenuation was associated with the patient's risk of irAEs [17]. Additionally, Yamaguchi et al. reported that 55.6% of patients with usual interstitial pneumonia developed ICI-induced pneumonitis [18]. In our case, it is possible that ICI use could have been avoided because slight ground-glass opacities remained after treatment. However, the patient did not tolerate chemotherapy because of her poor performance status, and the tumor had high PD-L1 expression. Therefore, ICI monotherapy was selected as the second-line treatment.

There is no well-established treatment protocol for patients with BO. An international clinical practice guideline suggested avoiding the use of sustained high-dose systemic corticosteroids because of the lack of proven benefits and the potential for serious adverse effects [19]. However, a meta-analysis showed that azithromycin improved the FEV_1_ of patients with BO after lung transplantation [20]. For the previous case of ICI-associated BO, oral corticosteroids, inhaled corticosteroids, long-acting bronchodilators, and azithromycin were prescribed. However, as in our case, the patient's lung function did not considerably improve. Cancer is not an indication for lung transplantation. Therefore, improving the symptoms and respiratory function of patients with ICI-associated BO remains challenging.

## 4 Conclusion

BOS after ICI treatment is severely toxic to the lungs and difficult to improve with medications. Although ICI-associated BOS is extremely rare, clinicians should know that it can occur during ICI administration.

## Declaration of competing interests

The authors have no conflicts of interest to declare.

## Funding sources

This research did not receive any specific grant from funding agencies in the public, commercial, or not-for-profit sectors.

## References

[1] Borghaei H., Paz-Ares L., Horn L., Spigel D.R., Steins M., Ready N.E., Chow L.Q., Vokes E.E., Felip E., Holgado E., Barlesi F., Kohlhäufl M., Arrieta O., Burgio M.A., Fayette J., Lena H., Poddubskaya E., Gerber D.E., Gettinger S.N., Rudin C.M., Rizvi N., Crinò L., Blumenschein G.R., Antonia S.J., Dorange C., Harbison C.T., Graf Finckenstein F., Brahmer J.R. (2015). Nivolumab versus docetaxel in advanced nonsquamous non-small-cell lung cancer. N. Engl. J. Med..

[2] Sears C.R., Peikert T., Possick J.D., Naidoo J., Nishino M., Patel S.P., Camus P., Gaga M., Garon E.B., Gould M.K., Limper A.H., Montgrain P.R., Travis W.D., Rivera M.P. (2019). Knowledge gaps and research priorities in immune checkpoint inhibitor-related pneumonitis. An official American thoracic society research statement. Am. J. Respir. Crit. Care Med..

[3] Postow M.A. (2015). Managing immune checkpoint-blocking antibody side effects, American Society of Clinical Oncology educational book. American Society of Clinical Oncology. Annual Meeting.

[4] Reck M., Rodríguez-Abreu D., Robinson A.G., Hui R., Csőszi T., Fülöp A., Gottfried M., Peled N., Tafreshi A., Cuffe S., O'Brien M., Rao S., Hotta K., Leiby M.A., Lubiniecki G.M., Shentu Y., Rangwala R., Brahmer J.R. (2016). Pembrolizumab versus chemotherapy for pd-l1-positive non-small-cell lung cancer. N. Engl. J. Med..

[5] Herbst R.S., Baas P., Kim D.W., Felip E., Pérez-Gracia J.L., Han J.Y., Molina J., Kim J.H., Arvis C.D., Ahn M.J., Majem M., Fidler M.J., de Castro G., Garrido M., Lubiniecki G.M., Shentu Y., Im E., Dolled-Filhart M., Garon E.B. (2016). Pembrolizumab versus docetaxel for previously treated, PD-L1-positive, advanced non-small-cell lung cancer (KEYNOTE-010): a randomised controlled trial. Lancet.

[6] Aguilar P.R., Michelson A.P., Isakow W. (2016). Obliterative bronchiolitis, Transplantation.

[7] Boehler A., Estenne M. (2003). Post-transplant bronchiolitis obliterans. Eur. Respir. J..

[8] Estenne M., Maurer J.R., Boehler A., Egan J.J., Frost A., Hertz M., Mallory G.B., Snell G.I., Yousem S. (2002). Bronchiolitis obliterans syndrome 2001: an update of the diagnostic criteria. J. Heart Lung Transplant..

[9] Blanchard A., Bouchard N. (2019). Pembrolizumab-induced obstructive bronchiolitis in a patient with stage IV non-small-cell lung cancer. Curr. Oncol..

[10] Delaunay M., Cadranel J., Lusque A., Meyer N., Gounant V., Moro-Sibilot D., Michot J.M., Raimbourg J., Girard N., Guisier F., Planchard D., Metivier A.C., Tomasini P., Dansin E., Pérol M., Campana M., Gautschi O., Früh M., Fumet J.D., Audigier-Valette C., Couraud S., Dalle S., Leccia M.T., Jaffro M., Collot S., Prévot G., Milia J., Mazieres J. (2017). Immune-checkpoint inhibitors associated with interstitial lung disease in cancer patients. Eur. Respir. J..

[11] Ebert M.P., Meindl-Beinker N.M., Gutting T., Maenz M., Betge J., Schulte N., Zhan T., Weidner P., Burgermeister E., Hofheinz R., Vogel A., Angermeier S., Bolling C., de Wit M., Jakobs R., Karthaus M., Stocker G., Thuss-Patience P., Leidig T., Gaiser T., Kather J.N., Haertel N. (2022). Second-line therapy with nivolumab plus ipilimumab for older patients with oesophageal squamous cell cancer (RAMONA): a multicentre, open-label phase 2 trial, the lancet. Healthy longevity.

[12] Barker A.F., Bergeron A., Rom W.N., Hertz M.I. (2014). Obliterative bronchiolitis. N. Engl. J. Med..

[13] Jagasia M.H., Greinix H.T., Arora M., Williams K.M., Wolff D., Cowen E.W., Palmer J., Weisdorf D., Treister N.S., Cheng G.S., Kerr H., Stratton P., Duarte R.F., McDonald G.B., Inamoto Y., Vigorito A., Arai S., Datiles M.B., Jacobsohn D., Heller T., Kitko C.L., Mitchell S.A., Martin P.J., Shulman H., Wu R.S., Cutler C.S., Vogelsang G.B., Lee S.J., Pavletic S.Z., Flowers M.E. (2015). National Institutes of Health consensus development project on criteria for clinical trials in chronic graft-versus-host disease: I. The 2014 diagnosis and staging working group report. Biol. Blood Marrow Transplant..

[14] West E.E., Lavoie T.L., Orens J.B., Chen E.S., Ye S.Q., Finkelman F.D., Garcia J.G., McDyer J.F. (2006). Pluripotent allospecific CD8+ effector T cells traffic to lung in murine obliterative airway disease. Am. J. Respir. Cell Mol. Biol..

[15] Wu Q., Gupta P.K., Suzuki H., Wagner S.R., Zhang C., O W.C., Fan L., Kaplan M.H., Wilkes D.S., Shilling R.A. (2015). CD4 T cells but not Th17 cells are required for mouse lung transplant obliterative bronchiolitis. Am. J. Transplant..

[16] Pain M., Bermudez O., Lacoste P., Royer P.J., Botturi K., Tissot A., Brouard S., Eickelberg O., Magnan A. (2014). Tissue remodelling in chronic bronchial diseases: from the epithelial to mesenchymal phenotype. Eur. Respir. Rev..

[17] Suazo-Zepeda E., Bokern M., Vinke P.C., Hiltermann T.J.N., de Bock G.H., Sidorenkov G. (2021). Risk factors for adverse events induced by immune checkpoint inhibitors in patients with non-small-cell lung cancer: a systematic review and meta-analysis. Cancer Immunol. Immunother..

[18] Yamaguchi T., Shimizu J., Oya Y., Watanabe N., Hasegawa T., Horio Y., Inaba Y., Fujiwara Y. (2022). Risk factors for pneumonitis in patients with non-small cell lung cancer treated with immune checkpoint inhibitors plus chemotherapy: a retrospective analysis. Thoracic cancer.

[19] Meyer K.C., Raghu G., Verleden G.M., Corris P.A., Aurora P., Wilson K.C., Brozek J., Glanville A.R. (2014). An international ISHLT/ATS/ERS clinical practice guideline: diagnosis and management of bronchiolitis obliterans syndrome. Eur. Respir. J..

[20] Kingah P.L., Muma G., Soubani A. (2014). Azithromycin improves lung function in patients with post-lung transplant bronchiolitis obliterans syndrome: a meta-analysis. Clin. Transplant..

